# Landscape of Prognostic m6A RNA Methylation Regulators in Hepatocellular Carcinoma to Aid Immunotherapy

**DOI:** 10.3389/fcell.2021.669145

**Published:** 2021-08-05

**Authors:** Qianhui Xu, Hao Xu, Rongshan Deng, Nanjun Li, Ruiqi Mu, Zhixuan Qi, Yunuo Shen, Zijie Wang, Jingchao Wen, Jiaxin Zhao, Di Weng, Wen Huang

**Affiliations:** ^1^The Second Affiliated Hospital and Yuying Children’s Hospital of Wenzhou Medical University, Wenzhou, China; ^2^Zhejiang University School of Medicine, Hangzhou, China

**Keywords:** m6A RNA methylation regulators, hepatocellular carcinoma, prognostic value, tumor immune microenvironment, immune checkpoint blockade, The Cancer Genome Atlas

## Abstract

**Background:** Hepatocellular carcinoma (HCC) is the sixth most common malignancy with a high mortality worldwide. N6-methyladenosine (m6A) may participate extensively in tumor progression.

**Methods:** To reveal the landscape of tumor immune microenvironment (TIME), ESTIMATE analysis, ssGSEA algorithm, and the CIBERSORT method were used. Taking advantage of consensus clustering, two different HCC categories were screened. We analyzed the correlation of clustering results with TIME and immunotherapy. Then, we yielded a risk signature by systematical bioinformatics analyses. Immunophenoscore (IPS) was implemented to estimate the immunotherapeutic significance of risk signature.

**Results:** The m6A-based clusters were significantly correlated with overall survival (OS), immune score, immunological signature, immune infiltrating, and ICB-associated genes. Risk signature possessed robust prognostic validity and significantly correlated with TIME context. IPS was employed as a surrogate of immunotherapeutic outcome, and patients with low-risk scores showed significantly higher immunophenoscores.

**Conclusion:** Collectively, m6A-based clustering subtype and signature was a robust prognostic indicator and correlated with TIME and immunotherapy, providing novel insight into antitumor management and prognostic prediction in HCC.

## Introduction

Hepatocellular carcinoma (HCC) characterized by high mortality is one of the most common global malignancies (Bray et al., 2018; Forner et al., 2018; Yang et al., 2019) with an estimated 841,080 newly added tumor cases and an almost 781,631 HCC-related mortality documented in 2018 (Bray et al., 2018). Great progression has been reached in the early diagnosis, clinical management, and prognosis supervision of HCC due to recent advances in various technological applications; however, the clinical outcome remains dismal (Grandhi et al., 2016; Song et al., 2017). The 5-year prognosis remains very poor given the frequent incidence of relapse and extrahepatic metastasis (Llovet et al., 2016). Currently, available prognosis monitoring indicators like alpha-fetoprotein (AFP) showed limited precision for prognostic prediction for HCC (Agopian et al., 2017; Lou et al., 2017; Ahn et al., 2020). Due to the risk of tumor seeding, liver biopsy is not extended widely though it is able to reveal specimen biology (Zhu and Hoshida, 2018; Xu et al., 2021a). Besides, the high heterogeneity of HCC greatly weakened the therapeutic effects and makes prediction of clinical outcome considerably sophisticated (Forner et al., 2018; Zhang et al., 2019b). The regulation of the immunological network plays a central role in the response to treatment and tumor progression of HCC (Nishida and Kudo, 2017). HCC-inducing lymphotoxin-α/β produced by CD8+ T cells promoted development of tumor and may play vital roles in tumor surveillance (Finkin et al., 2015). Experimental evidence showed that CD4+ T cell depletion was linked to HCC promotion (Ma et al., 2016). Due to the promotion of angiogenesis of inflammatory monocytes, CCL2 and CCR2 may be promising therapeutic targets of HCC (Li X. et al., 2017). Immune checkpoint blockade (ICB) immunotherapy has yielded great therapeutic effects in a wide variety of malignancies due to its precision and fewer side effects. Preclinical trial results showed that about 20% of patients benefited from ICB immunotherapy, indicating that immune checkpoint inhibitors may be conducive to HCC clinical management (Cheng et al., 2019). It is therefore imperative to screen robust and stable predictors to enhance the prognostic precision of HCC patients. Hence, the most effective tactic for the precise prognostic prediction of how a given malignancy will respond to immunotherapy or how clinical course will develop may be one derived from molecular risk distribution, identifying tumor patients based on particular biomarker signatures, generating an individualized program to improve efficacy accordingly.

*N6*-methyladenosine (m6A), the most prevalent type of modification on mRNA, refers to the methylation modification at the sixth N atom of adenine (Wang et al., 2017; Du et al., 2019). The level of m6A methylation depends on m6A RNA methylation regulator expression level in eukaryotic cells. The m6A modification can be reversed and is manipulated by intracellular binding proteins (“readers”), demethylases (“erasers”), and methyltransferases (“writers”) (Meyer and Jaffrey, 2014; Liu et al., 2017). Dysregulated m6A methylation levels serve as essential players in various physiological and pathological processes, such as microRNA (miRNA) editing, immune regulation, and tumor progression (Chen et al., 2015; Cui et al., 2017; Li H. et al., 2017). Nishizawa et al. (2018) pointed out that YTHDF1 is significantly overexpressed in colorectal cancer samples relative to adjacent normal specimens, and closely correlated to pathological stage. Taketo et al. (2018) revealed that the low expression level of METTL3 makes pancreatic cancer cells sensitive to radiotherapy and antitumor treatment. Emerging studies have demonstrated that dysregulated m6A modification level and its modulators are significantly linked to HCC tumorigenesis and development (Ma et al., 2017; Yang et al., 2017; Chen et al., 2018; Rong et al., 2019; Zhong et al., 2019). However, the relationships between m6A methylation modulators and tumor immune microenvironment (TIME) and ICB immunotherapy of HCC remain elusive.

In this work, the potential players of m6A RNA methylation modulators in prognosis, TIME, and ICB immunotherapy of HCC were our primary concerns. Clustering subgroups and risk signature for m6A-related genes were developed to enhance prognostic risk classification and facilitate identification of candidate promising therapeutic targets for clinical strategies in HCC. Then, the correlation of clustering subtypes and risk signature with immune infiltration and immune-related scores were comprehensively performed to further investigate the underlying influence of m6A RNA methylation regulators upon TIME characterization. Furthermore, the response to immunotherapy in patients with different risk scores was predicted to contribute novel insights into management decision-making for HCC immunotherapy. Finally, the biological role of ZC3H13 was analyzed in the clinical outcome and progress of HCC.

## Materials and Methods

### Public Data Collection

RNA-sequencing transcriptomic data in the fragments per kilobase per million (FPKM) format and the clinical information of HCC cases were obtained from The Cancer Genome Atlas (TCGA) portal^1^ for subsequent analysis. All analyses were performed based on the publication guidelines of TCGA. After patients lacking complete genomics or clinical data were excluded, a total of 370 HCC specimens and 50 normal hepatic tissue cases were employed for further analysis. The LIRI dataset including 231 HCC samples and 202 normal tissues from the ICGC database was employed as the external testing group. The corresponding expression profiling information and the clinical data were downloaded from the ICGC^2^. All data were publicly available and open access, so it was unnecessary to obtain Ethics Committee approval. Data were processed in accordance with the NIH TCGA human subject protection^3^ and related data access policies.

### Expression Pattern of m6A RNA Methylation Regulators

The expression data of 21 m6A RNA methylation regulators (ALKBH5, EIF3A, FTO, HNRNPA2B1, HNRNPC, IGF2BP1, IGF2BP2, IGF2BP3, KIAA1429, METTL14, METTL16, METTL3, RBM15, RBM15B, WTAP, YTHDC1, YTHDC2, YTHDF1, YTHDF2, YTHDF3, and ZC3H13) were extracted for further analysis based on previous research (Batista, 2017; Dai et al., 2018; Chai et al., 2019). Subsequently, R package “Limma” was used to analyze the expression of 21 m6A regulators in the tumor specimen vs. the normal counterpart. Statistical significance threshold was set as follows: absolute log2 fold change (FC) > 1 and *p*-value < 0.05. Subsequently, a boxplot was employed to present these m6A regulators’ expression level in tumor tissues and normal samples. Pearson correlation analysis was carried out *via* using the “corrplot” package to reveal the relationship between m6A regulators. To further elucidate the m6A regulators’ expression results from the standpoint of fundamental biology, we conducted Gene ontology (GO) annotation on m6A-related genes differentially expressed between tumor sample and normal tissue.

### Landscape of Immune Cell Infiltration in a Tumor Immune Environment

Taking advantage of the CIBERSORT package^4^, the gene expression information of TCGA and ICGC HCC cohorts was analyzed to obtain a fraction matrix of TICs, which estimates the cellular composition of immunity (Newman et al., 2015). To explore the correlation of TICs with clinical variables, age, gender, grade and stage were employed. To explore the prognostic predictive significance of TICs, Kaplan–Meier curves analysis was performed between the low- and high-fraction group.

### Consensus Clustering of HCC Cases

To functionally comprehend the biological significance of the m6A RNA methylation regulators in HCC, the “ConsensusClusterPlus” package was employed to stratify the HCC samples into two distinct subgroups, with a hierarchical agglomerative consensus, based on the m6A RNA modification regulator expression information. Unsupervised clustering methods utilize the proportion of ambiguous clustering (PAC) to verify different expression patterns between two different HCC clusters. Next, the survival package was utilized to determine the differential prognosis of two distinct subtypes based on the Kaplan–Meier method. Analysis focusing on the correlation of cluster 1/2 with clinicopathological features (i.e., age and gender) was performed *via* the chi-square test. A single-sample gene set enrichment analysis (ssGSEA) was performed to assign the enrichment activity of 29 immune function-associated pathways using the “GSEAbase” R package. Additionally, the R package “ESTIMATE” was applied to estimate the extent of infiltrating cells, namely, immune cells and stromal cells, and level of tumor purity, which could validate significant distinct characterization of TIME. Then, the fraction of 22 immune cellular subtypes for each tumor specimen was calculated through cell-type identification by estimating relative subsets of RNA transcripts (CIBERSORT; see footnote 4). Finally, the expression levels of 47 ICB-related genes (e.g., CTLA4) of each tumor tissue were detected.

### Establishment of Prognostic Risk Signature

The candidate m6A regulators significantly correlated with prognosis (*p* < 0.05) were screened by using univariate COX regression on the expression level of 21 m6A regulators. Subsequently, a gene’s risk coefficient was computed by employing LASSO regression algorithm with the “glment” package after the elimination of highly correlated genes. Next, six m6A regulators were identified and employed to assemble a prognostic risk signature. The risk score of each sample was obtained using the following equation: risk score = sum of risk coefficients ^∗^ expression level of m6A regulators.

### Validation of Prognostic Risk Signature

According to the median risk score, HCC cases were assigned into low-/high-risk subgroups. Kaplan–Meier survival curves with “survival” R package were analyzed. Next, the time-dependent receiver operating characteristic (ROC) curves were plotted to validate the prognostic performance. Then, univariate and multivariate Cox regression analyses were performed to confirm the independent prognostic predictive factor. R package “pheatmap” was employed to correlate clinicopathological variables with the risk score, and differences in clinical data between high- and low-risk sets were identified by the chi-square test. To validate the external reliability of this m6A-based prognostic signature, the ICGC LIRI dataset was extracted as the validation group. Risk scores of LIRI patients were obtained in the same equation as mentioned above. HCC samples were separated into low- and high-risk subgroups after the median risk score serving as the cutoff point. Next, Kaplan–Meier survival analysis, ROC curve, and correlation between risk score and clinical feature were employed to estimate the prognosis predictive performance.

### Risk Score in Characterization of TIME

To exhibit the comprehensive landscape of TIME in low-/high-risk groups in both the TCGA and ICGC HCC cohort, we conducted several analyses. The estimate score, stromal score, immune score, and tumor purity of each case were calculated with the ESTIMATE algorithm *via* the R package “estimate” to reveal overall TIME characterization of two different risk score groups. Besides, the R package GSEABase of 29 immune-correlated enrichments was employed to further identify the difference in immunity-related response between different risk subgroups. Subsequently, the R package “CIBERSORT” was employed to estimate subpopulations of 22 immune cells in TIME.

### Development of Prognostic Nomogram

To estimate the prognostic prediction of the risk model, age, gender, WHO grade and clinical stage, and time-dependent ROC curves of 1/2/3-year OS were analyzed to compute the area under the curve (AUC) values (Blanche et al., 2013). To provide a scoring system to predicting prognosis quantitatively, a prognostic nomogram that consists of a risk score and clinical variables was established to assess 1–, 2–, and 3-year OS possibility. Additionally, the calibration curve, which could validate the prognostic value of the as-constructed nomogram, was analyzed.

### Role of Risk Signature in Biological Processes

Gene set enrichment analysis (GSEA) was conducted to functionally understand biological players of the as-constructed risk signature in HCC development. We analyzed the gene sets of “c2.cp.kegg.v7.2.symbols.gmt [Curated]” from the Molecular Signatures Database through GSEA (Subramanian et al., 2005). To achieve a normalized enrichment score for each analysis, gene set permutations with 1,000 times were carried out. A nominal *p* < 0.05 and FDR *q* < 0.05 were retained as significant results.

### ZC3H13 in the Context of TIME

Immune infiltration data consisting of immune cell fractions (i.e., B cells, CD4 + T cells, CD8 + T cells, dendritic cells, macrophages, and neutrophils) were obtained from Tumor Immune Estimation Resource (TIMER)^5^. The correlation of prognostic risk signature with immune cell infiltration was used to investigate whether our risk model plays a crucial role in the formation of complexity and diversity of TIME. Besides, the relationship between ZC3H13 expression level and immune infiltration was correlated and analyzed *via* TIMER portal.

### Prediction of Patients’ Response to Immunotherapy

Based on published articles, ICB-relatedgenes expression level may be correlated with treatment responses of immune checkpoint inhibitors(Goodman et al., 2017). In this study, six genes of ICB therapy—cytotoxic T-lymphocyte antigen 4 (CTLA-4), programmed death 1 (PD-1, also known as PDCD1), programmed death ligand 1 (PD-L1, also known as CD274), programmed death ligand 2 (PD-L2, also known as PDCD1LG2), T-cell immunoglobulin domain and mucin domain-containing molecule-3 (TIM-3, also known as HAVCR2), and indoleamine 2,3-dioxygenase 1 (IDO1)—were investigated (Kim et al., 2017; Nishino et al., 2017; Zhai et al., 2018). To reveal the potential role of risk score in ICB treatment, risk signature was correlated with the expression level of six ICB genes. Furthermore, the association between the expression level of ZC3H13 and that of key immunological checkpoints (i.e., PDCD1, PDCD1LG2, CD274, CTLA4, IDO1, and HAVCR2) was analyzed. Furthermore, the expression levels of 47 ICB-related genes (e.g., PDCD1) were comprehensively determined.

Immunophenoscore (IPS) refers to four main parts (effector cells, immunosuppressive cells, MHC molecules, and immunomodulators) that determine immunogenicity and is calculated without bias using machine learning methods. The IPS (range, 0–10) is calculated based on the gene expression in representative cell types. It has been verified that IPS could predict the patients’ response to immunotherapy (Charoentong et al., 2017). The IPSs of HCC patients were downloaded from The Cancer Immunome Atlas (TCIA)^6^.

### Distribution of ZC3H13 Based on Single-Cell RNA Sequencing Analysis

To explore the potential players of ZC3H13 in TIME, single-cell transcriptome sequencing data GSE140228 were employed (Zhang et al., 2019a), which are the transcriptome data of CD45 + immune cells made by Zemin Zhang’s team for HCC patients. The researchers uploaded the hepatic carcinoma single-cell RNA sequencing data of the study to an interactive website^7^ to facilitate researcher in-depth exploration of related fields. In this work, 10 × Genomics sequencing data were used to analyze the expression of ZC3H13 in tumor, adjacent liver, hepatic lymph node, blood, and ascites, and compare the expression level of ZC3H13 in immune cell subpopulations.

### Experimental Validation

QSG-7701 (human hepatic cell line) and four human HCC cell lines (Hep-3B cells, MHCC-97H cells, Huh7 cells, and HCC-LM3 cells) were obtained from the Cell Bank of the Type Culture Collection of the Chinese Academy of Sciences, Shanghai Institute of Biochemistry and Cell Biology. The cell lines were all cultured in Dulbecco’s minimum essential media (DMEM) plus 10% fetal bovine serum (FBS; Invitrogen, Carlsbad, CA, United States). These different cell lines were subjected to quantitative real-time polymerase chain reaction (qRT-PCR). qRT-PCR was analyzed as described previously (Xu et al., 2021c). All samples were analyzed in triplicate. Glyceraldehyde-3-phosphate dehydrogenase (GAPDH) levels were used as the endogenous control and the relative expression of ZC3H13 was calculated using the 2^–ΔΔ^ ^CT^ method. The sequences of primers used for PCR were as follows: ZC3H13, 5′-CGGACAGTGATGCCTACAACAGTG-3′ (forward) and 5′-TGAGGTGCGAGGGACTAAGAGAAC-3′ (reverse); and GAPDH, 5′-CAGGAGGCATTGCTGATGAT-3′ (forward) and 5′-GAAGGCTGGGGCTCATTT-3′ (reverse).

### Statistical Analysis

The expression level of m6A regulators was compared using one-way ANOVA in tumor tissue versus normal sample, while *t*-tests were analyzed to identify the differential expression levels of m6A regulators for age, gender, clinicopathological stage, and TNM status. Overall survival (OS) refers to the interval from the date of diagnosis to the date of death. Survival curves were analyzed using the Kaplan–Meier log rank test. Subgroups, risk scores, clinical variables, immune cell infiltration, and immune checkpoints were correlated with Pearson correlation test. CIBERSORT algorithm results with *p* < 0.05 were employed for further analysis. Univariate and multivariate COX regression were analyzed to validate the independent prognosis predictive performance of risk signature. *p* < 0.05 was considered as statistically significant. R software (version 3.6.3) was utilized for all statistical analyses.

## Results

### Analysis of m6A Regulator Expression Pattern in HCC

**FIGURE 1:**
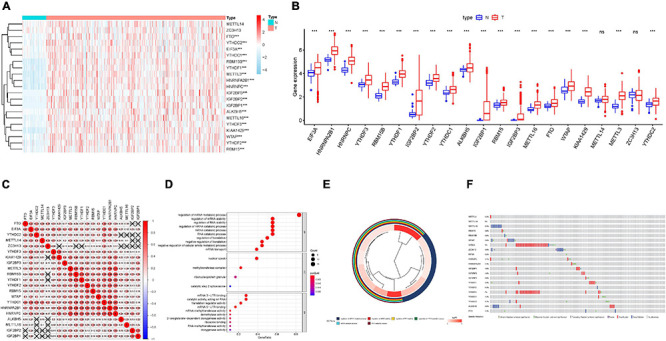
Expression patterns of m6A RNA modification regulators in HCC. **(A)** The heatmap of m6A regulator expression levels in each case. The color from red to blue shows a trend from high expression to low expression. **(B)** The boxplot visualizes the abnormally expressed m6A regulators in tumor. N represents normal specimen and T represents tumor specimen. **(C)** Broad co-expression correlation among the 21 m6A RNA modification regulators in HCC. “ × ” means *p* > 0.05. **(D)** Barplot and **(E)** clusterplot of gene ontology (GO) analyses of differentially expressed m6A-related genes in tumor. **(F)** Genetic alteration was analyzed *via* cBioPortal database. The asterisks represented the statistical *p* value (***P < 0.001).

The landscape of 21 m6A regulators’ expression pattern (Table 1) was comprehensively analyzed in tumor specimens and paired normal samples from the TCGA HCC cohort. We observed that the expression levels of most m6A regulators were significantly distinct between tumor tissues and adjacent samples (Figures 1A,B). Additionally, further validation was analyzed in the ICGC-LIRI-JP dataset (Supplementary Figure 1A). Concretely, m6A-related genes, including ALKBH5, EIF3A, FTO, HNRNPA2B1, HNRNPC, IGF2BP1, IGF2BP2, IGF2BP3, KIAA1429, METTL16, METTL3, RBM15, RBM15B, WTAP, YTHDC1, YTHDC2, YTHDF1, YTHDF2, and YTHDF3 (all *p* < 0.001), were dramatically higher in HCC specimens relative to adjacent normal liver samples. However, we did not find a statistically significant distinction in terms of METTL14 as well as ZC3H13 (*p* = 0.06 and 0.83, respectively). To further elucidate the inherent relationship, we analyzed the correlation among these m6A regulators. Notably, the intrinsic connection between HNRNPC and HNRNPA2B1 is the most significant one presented in Figure 1C. To further understand the m6A regulators’ expression patterns from the standpoint of biological procedures, we employed GO annotation on genes whose expression level was abnormally regulated in HCC tissues. Figures 1D,E show that upregulated m6A-related genes were mainly enriched in mRNA-related regulatory processes, including regulation of mRNA stability and regulation of mRNA metabolic process.

**TABLE 1:** The basic information of the included m6A RNA methylation regulators.

**Gene_name**	**The role in m6A**	**Ensemble**	**Location**
ALKBH5	Eraser	ENSG00000091542	Chromosome 17, NC_000017.11
EIF3A	Reader	ENSG00000107581	Chromosome 10, NC_000010.11
FTO	Eraser	ENSG00000140718	Chromosome 16, NC_000016.10
HNRNPA2B1	Reader	ENSG00000122566	Chromosome 7, NC_000007.14
HNRNPC	Reader	ENSG00000092199	Chromosome 14, NC_000014.9
IGF2BP1	Reader	ENSG00000159217	Chromosome 17, NC_000017.11
IGF2BP2	Reader	ENSG00000073792	Chromosome 3, NC_000003.12
IGF2BP3	Reader	ENSG00000016797	Chromosome 7, NC_000007.14
KIAA1429	Writer	ENSG00000164944	Chromosome 8, NC_000008.11
METTL14	Writer	ENSG00000145388	Chromosome 4, NC_000004.12
METTL16	Writer	ENSG00000127804	Chromosome 17, NC_000017.11
METTL3	Writer	ENSG00000165819	Chromosome 14, NC_000014.9
RBM15	Writer	ENSG00000162775	Chromosome 1, NC_000001.11
RBM15B	Writer	ENSG00000259956	Chromosome 3, NC_000003.12
WTAP	Writer	ENSG00000146457	Chromosome 6, NC_000006.12
YTHDC1	Reader	ENSG00000083896	Chromosome 4, NC_000004.12
YTHDC2	Reader	ENSG00000047188	Chromosome 5, NC_000005.10
YTHDF1	Reader	ENSG00000149658	Chromosome 20, NC_000020.11
YTHDF2	Reader	ENSG00000198492	Chromosome 1, NC_000001.11
YTHDF3	Reader	ENSG00000185728	Chromosome 8, NC_000008.11
ZC3H13	Writer	ENSG00000123200	Chromosome 13, NC_000013.11

### Landscape of m6A Regulator Mutation in HCC

Genetic alteration information of m6A regulators was explored employing the TCGA HCC cohort on the cBioPortal database to uncover the potential influence of genetic alteration upon the corresponding gene expression (Figure 1F). On the whole, we found that VIRMA had the highest alternation proportion and exhibited 9% genetic alteration, and the most common alteration manner was amplification.

### Immune Cell Infiltration Subsets in a Tumor Immune Environment of HCC

**FIGURE 2:**
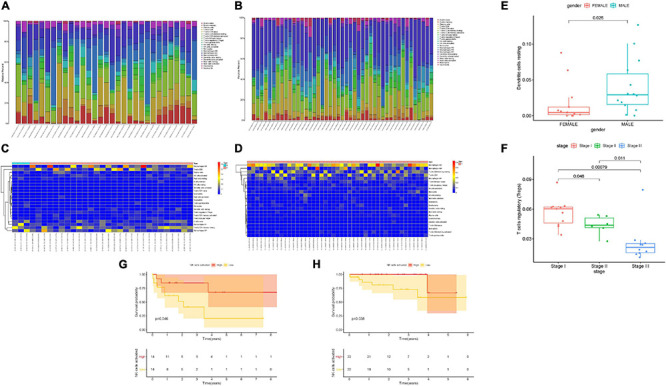
Landscape of immune cell infiltration in tumor immune environment of HCC. Subpopulation of 22 immune cell subtypes in the TCGA cohort **(A)** and ICGC cohort **(B)**. Proportional heatmap of the 22 TICs in each patient of the TCGA cohort **(C)** and ICGC cohort **(D)**. **(E)** Infiltrating resting dendritic cell was significantly associated with patient gender. **(F)** Infiltration of regulatory T cells significantly decreased with advanced stages. Activated NK cell infiltration significantly correlated with better prognosis in both the TCGA cohort **(G)** and ICGC cohort **(H)**.

To assess the composition of 22 TIC types, the CIBERSORT algorithm was employed in not only the TCGA dataset but also the ICGC dataset. The overall fraction of immune cells in HCC is shown in Figures 2A,B. The highest proportion of TICs was resting CD4 memory T cells in the TCGA cohort, whereas naive B cells accounted for the most abundant infiltrating immune cells in the ICGC cohort, suggesting that activated immune cells mediated in antitumor response may exert an opposing player in HCC tumorigenesis and progression. Figures 2C,D show the distributions of 22 immune cells’ proportion together with HCC patients. To elucidate the clinical significance of TICs, we correlated components of 22 TICs with clinicopathological characteristics. We found that the distribution of resting dendritic cells had a close correlation with patient gender (Figure 2E). The composition of regulatory T cells reduced significantly with advanced clinical stage (all *p* < 0.05, Figure 2F), indicating that regulatory T cells might serve as a suppressing role in HCC development. To estimate the prognostic predictive performance of TICs, we analyzed patient prognosis based on distinct TICs fraction. Taking advantage of the Kaplan–Meier method, abundance of activated NK cells was significantly correlated with better prognosis in the TCGA cohort (Figure 2G, *p* = 0.046). Likewise, activated NK cells had a close association with longer OS in the ICGC cohort (Figure 2H, *p* = 0.038). These results suggested that Tregs and activated NK cells may serve as non-negligible players in the antitumor response of HCC.

### Consensus Clustering in Prognosis, Clinical Features, and TIME of HCC

**FIGURE 3:**
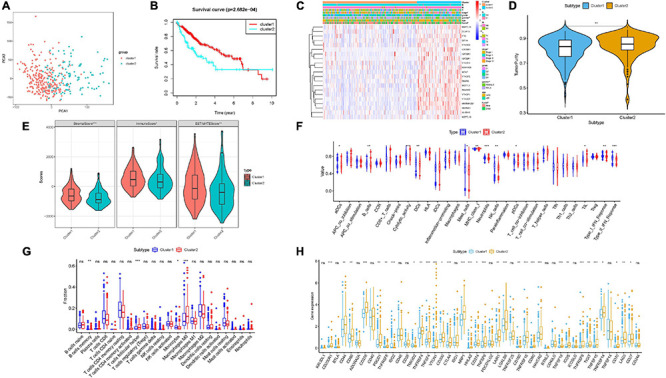
Consensus clustering based on the expression pattern of m6A regulators. **(A)** Principal component analysis of the total RNA expression profile. **(B)** Kaplan–Meier overall survival (OS) curves for the TCGA HCC cohort. **(C)** Heatmap together with clinical features of cluster 1/2. Blue represents downregulated expression and red represents upregulated expression. **(D,E)** The estimate score, stromal score, immune score, and tumor purity differ well between these two clusters. **(F)** Enrichment of immune-related signatures was significantly distinct between two HCC subtypes. **(G)** Comparison of infiltrating immune cell subtypes and levels between clusters 1 and 2. **(H)** Expression levels of 47 immune checkpoint blockade-related genes in two different subgroups. The asterisks represented the statistical *p* value (*P < 0.05; **P < 0.01; ***P < 0.001).

To better reveal the clinicopathological value of 21 m6A regulators, patients were clustered into two different subtypes according to the expression pattern of m6A regulators. According to similarities displayed in m6A modulators, we observed that *k* = 2 had optimal clustering stability. An increasing trend of the cumulative distribution function (CDF) value was regarded as an indicator of excellent clustering (Supplementary Figures 1B–D). To further support the result of consensus clustering, principal component analysis (PCA) was performed, which showed that cluster 1/2 were non-overlapping and differentiated well (Figure 3A). Subsequently, OS time of cluster 2 was shorter than cluster 1 in Kaplan–Meier analysis (Figure 3B, *p* = 2.682e–04). Then, differences in the clinicopathological variables between the two subgroups were investigated. As a result, most m6A-related genes were remarkably upregulated in cluster 2 relative to cluster 1. In addition, Figure 3C shows that cluster 2 possessed significant correlation with female gender and advanced clinicopathological stage (both *p* < 0.05). Therefore, these results suggested that the expression pattern of m6A modulators may act as key regulators in HCC malignancy.

To elucidate the correlation of m6A regulators with TIME of HCC, we analyzed the immune infiltration type and extent and calculated the corresponding immunoscore of cluster 1/2. We explored whether there was a distinction between two HCC subtypes regarding the immune score, estimate score, tumor purity, and stromal score. Our results showed that relative to cluster 1, cluster 2 obtained lower estimate, stromal, and immune scores (Figure 3E) but higher tumor purity (Figure 3D). The difference in immune-related signature between clusters 1 and 2 was that subtype 2 was closely correlated with higher aDCs and MHC class I, whereas subtype 1 experienced more neutrophils, B cells, pDCs, NK cells, DCs, mast cells, cytolytic activity, TIL, and Type I/II IFN response, indicating that the distinction of m6A regulators’ expression pattern significantly correlated with TIME characterization of HCC (Figure 3F and Supplementary Figure 1E). Supplementary Figure 1F shows immune-associated enrichment pathways with corresponding immune scores of each sample in cluster 1/2. Next, the differential subpopulation of infiltrating tumor immune cells between two different subtypes was identified. The results showed that cluster 1 had a higher abundance of monocytes and memory B cells, whereas infiltration of M0 macrophages and follicular helper T cells was remarkably lower (Figure 3G).

To further uncover the involvement of m6A regulators with ICB treatment, expression levels of 47 ICB-related genes were analyzed between two clusters. Compared with the cluster 1 group, expression levels of the majority of ICB-associated genes were dramatically higher in cluster 2 (i.e., PDCD1 and CTLA4; Figure 3H). Hence, the clustering results might contribute to reveal the complexity of TIME and predict ICB therapy outcome in HCC.

### Construction of Prognostic Risk Signature

To further explore the prognostic significance of m6A modulators, univariate Cox regression analysis on 21 m6A regulators’ expression levels was conducted. As a result, 14 out of 21 m6A regulators had a significant association with OS (*p* < 0.05, Supplementary Figure 2A). Notably, YTHDF2, YTHDF1, IGF2BP3, METTL3, RBM15B, HNRNPA2B1, KIAA1429, HNRNPC, WTAP, IGF2BP1, YTHDC1, RBM15, and IGF2BP2 were deemed unfavorable prognostic factors (all HRs > 1, Supplementary Figure 2A), whereas only ZC3H13 was regarded as a beneficial prognostic indicator (HR < 1, Supplementary Figure 2A). Then, LASSO algorithm was analyzed to identify m6A regulators with the most powerful prognosis predictive ability (Supplementary Figures 2B,C).

Finally, six m6A-related genes, namely, YTHDF1, YTHDF2, IGF2BP3, KIAA1429, METTL3, and ZC3H13, were recognized to constructed a m6A-based risk signature for HCC patients. Supplementary Figure 2D shows the corresponding coefficients.

The risk score of HCC patients was calculated using the following equation: risk score = (0.0262 ^∗^ expression level of YTHDF1) + (0.0577 ^∗^ expression level of YTHDF2) + (0.1192 ^∗^ expression level of IGF2BP3) + (0.027 ^∗^ expression level of KIAA1429) + (0.0795 ^∗^ expression level of METTL3) – (0.1018 ^∗^ expression level of ZC3H13).

Subsequently, each HCC patient obtained a corresponding risk score and was randomized into low-/high-risk subgroups based on the median threshold.

### Confirmation of Prognostic Risk Signature

**FIGURE 4:**
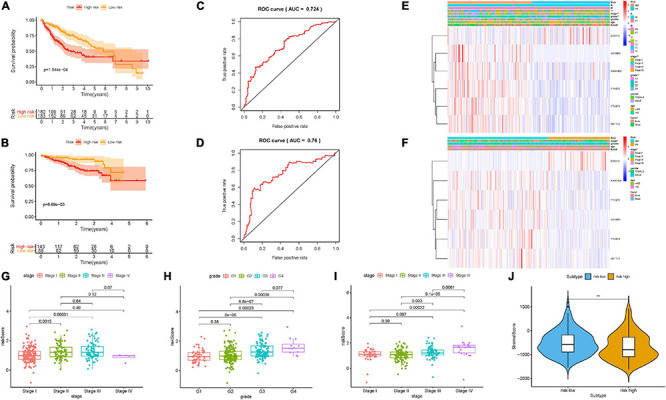
Construction of the prognostic risk signature based on m6A regulators. Kaplan–Meier curve analysis presenting differences in overall survival between the high-risk and low-risk groups in the TCGA cohort **(A)** and ICGC cohort **(B)**. ROC analysis of the risk scores for overall survival predictive significance in the TCGA cohort **(C)** and ICGC cohort **(D)**. The AUC was calculated for ROC curves, and sensitivity and specificity were calculated to assess score performance. Heatmap presents the distribution of clinical variables and the expression level of six m6A regulators in each patient in the TCGA cohort **(E)** and ICGC cohort **(F)**. **(G,H)** Risk score was significantly correlated with clinicopathological stage and clinical grade in the TCGA dataset. **(I)** Risk score had a significant correlation with clinicopathological stage in the ICGC cohort. **(J)** Comparison of tumor purity between these two subtypes. The asterisks represented the statistical *p* value (**P < 0.01).

Supplementary Figure 3A displays the distributions of six m6A regulators’ expression level with corresponding subgroups and patients. The allocations of dot pot and risk score of survival status in the TCGA-LIHC cohort highlighted that high-risk HCC samples experienced poorer prognosis (Supplementary Figures 3C,E). Additionally, survival analysis demonstrated that samples in the low-risk group presented significantly longer OS time than samples in the high-risk group (*p* = 1.544e–04; Figure 4A). ROC curve analysis was performed to estimate the prognostic predictive performance. AUC of risk score signature at 3-year survival times was 0.724, highlighting the great specificity and sensitivity of the prognostic value (Figure 4C). Moreover, results of univariate Cox regression showed that the hazard ratio (HR) of risk score was 3.713 (95% CI: 2.411–5.716; Supplementary Figure 3G). Corresponding results were discovered in multivariate Cox regression analysis (HR = 3.386, 95% CI: 2.168–5.290; Supplementary Figure 3I), indicating that risk score could act as an independent prognostic factor. Furthermore, the involvement of m6A-related genes with clinicopathological features was investigated and presented in the heatmap (Figure 4E). We observed that with advanced clinical stage (two out of six, *p* < 0.05, Figure 4G) and high pathological grade (most *p* < 0.05, Figure 4H), risk score was significantly elevated.

### Validation of Risk Prognostic Signature

To further estimate its external prognostic validity, we employed the ICGC dataset (LIRI) as an external testing group. The ICGC-LIRI cohort with 231 HCC samples was classified into low-risk and high-risk subgroups using the median threshold in the TCGA dataset. These results presented the distributions of m6A regulators’ expression patterns, risk score, and survival status in the external validation cohort (Supplementary Figures 3B,D,F) and the combination set (Supplementary Figures 4A–C). Likewise, survival curves showed that high-risk samples possessed significantly poorer prognosis relative to the low-risk group in the validation cohort (Figure 4B, *p* = 6.69e–03) and the combination set (Supplementary Figure 4D, *p* = 5.545e–05). The value of area under the ROC (AUC) was 0.76 in the external testing set (Figure 4D), suggesting the good prognostic performance of risk prognostic signature among different populations. Consistent with results in the training group, risk signature as a prognostic factor independently affected OS in both the validation group and the whole cohort (Supplementary Figures 3H,J, 4E,F). Subsequently, we plotted the heatmap to simultaneously present clinical relevance (Figure 4F). Notably, the higher the risk score, the more serious the clinical stage (most *p* < 0.05, Figure 4I).

### Correlation of Prognostic Risk Score With Characterization of TIME

**FIGURE 5:**
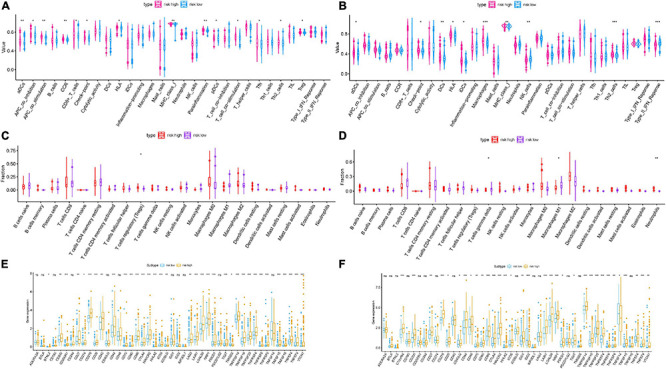
Correlation of prognostic risk score with TIME characterization of HCC. Distinction of enrichment of immune-related signatures between low-risk and high-risk group in the TCGA cohort **(A)** and ICGC cohort **(B)**. Difference in infiltrating immune cell subpopulations and levels between the low- and high-risk group in the TCGA cohort **(C)** and ICGC cohort **(D)**. Comparison of 47 immune checkpoint blockade-related gene expression levels in two risk score subgroups in the TCGA cohort **(E)** and ICGC cohort **(F)**. The asterisks represented the statistical *p* value (*P < 0.05; **P < 0.01; ***P < 0.001).

To reveal the potential roles of risk score in immune regulation, the correlation analyses of the risk score were performed with immune score, ssGSEA enrichment, and TIC abundance, and expression levels of 47 ICB-associated genes. It was discovered that samples with low risk experienced a higher stromal score than high-risk samples in the TCGA dataset but not the ICGC cohort (Figure 4J and Supplementary Figure 5B). Conversely, there was no significant difference in estimate score, tumor purity, and immune score (Supplementary Figures 5A,B). Combining the ssGSEA results of the two datasets, the infiltration of aDCs, Th2 cells, DCs, and some immune enrichments such as MCH class I expression, checkpoint, and HLA molecule expression level were significantly escalated with increased risk score (Figures 5A,B and Supplementary Figures 5C,D). Supplementary Figures 5E,F show the immune-associated enrichment of each sample with the corresponding immune score from two different datasets. The results of the CIBERSORT algorithm suggested that abundance of Tregs (regulatory T cells) was positively correlated with risk score in the TCGA dataset (Figure 5C), whereas ICGC patients with high risk presented fewer M1 macrophages, fewer gamma delta T cells, and more neutrophils relative to the low-risk group (Figure 5D). Subsequent correlation analysis showed that 25 of 47 (e.g., CTLA4) ICB-correlated genes were significantly overexpressed in high-risk samples (Figures 5E,F). These findings highlighted that m6A-based risk score may contribute a novel insight into the immunity regulatory network and further forecast immunotherapy outcome in HCC.

### Prognostic Significance of m6A-Based Risk Score in HCC

**FIGURE 6:**
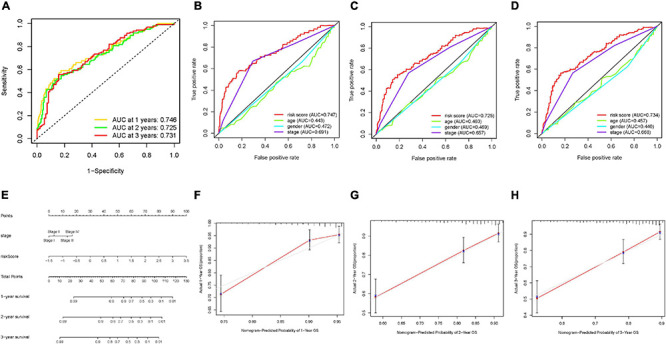
Validation of prognostic efficiency of m6A-based signature in HCC. **(A)** ROC analysis was employed to estimate the prediction value of the prognostic signature. **(B–D)** Areas under curves (AUCs) of the risk scores for predicting 1–, 2–, and 3-year overall survival time with other clinical characteristics. **(E)** Nomogram was assembled by age and risk signature for predicting survival of HCC patients. **(F)** One-year nomogram calibration curves of the combination of the TCGA and ICGC cohort. **(G)** Two-year nomogram calibration curves of the combination of the TCGA and ICGC cohort. **(H)** Three-year nomogram calibration curves of the combination of the TCGA and ICGC cohort.

Then, ROC curves were analyzed and the values of AUC for 1–, 2–, and 3-year OS were 0.746, 0.725, and 0.731, respectively, indicating good predictive accuracy (Figure 6A). To demonstrate risk score as the best prognostic factor among various clinical candidate variables, age, gender, clinical stage, and tumor grade were employed as the candidate prognostic indicators. These clinical variables were integrated to perform the AUC analysis for 1–, 2–, and 3-year OS, which showed that risk score experienced the highest value of AUC (Figures 6B–D). Subsequently, a prognostic nomogram including risk score and clinical stage was developed to predict clinical outcomes (Figure 6E). Age, gender, and tumor grade whose AUCs were less than 0.6 were rejected out of construction of the nomogram. In addition, calibration curves highlighted excellent prognostic prediction of the as-constructed nomogram (Figures 6F–H).

Next, stratification analysis was performed to examine whether risk score retained great prognostic performance when samples were assigned into various subgroups according to clinical features. Compared with low-risk samples, samples in the high-risk group experienced shorter OS time in the late- and early-stage subgroups (Supplementary Figures 6A,B). Likewise, risk score exhibited great prognostic significance for samples in the T1–2 or T3–4 category (Supplementary Figures 6C,D), male samples (Supplementary Figure 6E), samples in grades 1 and 2 (Supplementary Figure 6G), samples aged ≤ 65 years (Supplementary Figure 6I), samples with an N0 status (Supplementary Figure 6K), and samples with an M0 status (Supplementary Figure 6L). Meanwhile, it was discovered that the prognostic predictive ability of risk score was lost in female samples (Supplementary Figure 6F), grade 3–4 samples (Supplementary Figure 6H) or samples aged > 65 years (Supplementary Figure 6J). These findings indicated that it was an outstanding prognostic indicator in HCC patients.

### Functional Annotation of Prognostic Risk Signature

To further investigate the potential role of risk score mediated in HCC from the perspective of biological processes, GSEA was conducted in the low- and high-risk subgroups. The results of GSEA showed that the high-risk score was significantly enriched in pathways (i.e., prostatic cancer, non-small cell lung cancer, Wnt signal pathway, mTOR signal pathway, MAPK signal pathway, and the p53 signal pathway; Supplementary Figure 4G).

### Correlation of Risk Signature With Infiltrating Immune Cells

Furthermore, the correlation of the as-constructed risk score was explored with infiltrating immune cells in TIME. These results showed that risk signature presented significant positive correlation with the infiltration of B cells (*r* = 0.218; *p* = 2.752e–05), infiltration of CD4+ T cells (*r* = 0.200; *p* = 1.151e–04), infiltration of CD8+ T cells (*r* = 0.209; *p* = 5.891e–05), infiltration of dendritic cells (*r* = 0.305; *p* = 2.735e–09), infiltration of macrophages (*r* = 0.404; *p* = 8.609e–16), and infiltration of neutrophils (*r* = 0.349; *p* = 6.339e–12; Supplementary Figures 7A–F). Our findings provided strong evidence to validate that the m6A-based risk score experienced complex interactions with immune cell infiltration in HCC.

### Predicting Patients’ Clinical Outcome to Immunotherapy

**FIGURE 7:**
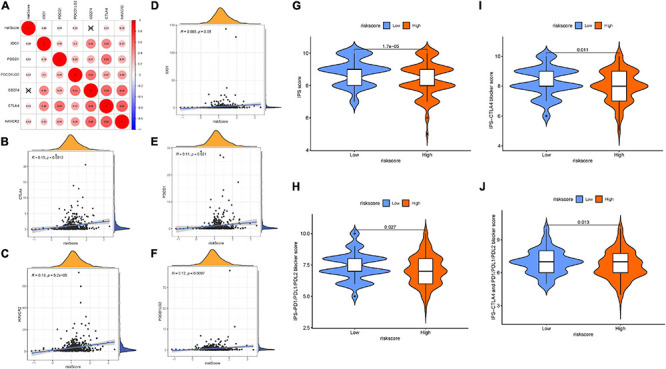
The estimation of two prognostic subtypes in immunotherapy response. Correlation between prognostic risk signature with crucial immune checkpoint genes. **(A)** Correlation analysis between immune checkpoint inhibitors (CD274, PDCD1, PDCD1LG2, CTLA4, HAVCR2, and IDO1) with prognostic risk signature. **(B)** Correlation between prognostic risk signature and CTLA4. **(C)** Correlation between prognostic risk signature and HAVCR2. **(D)** Correlation between prognostic risk signature and IDO1. **(E)** Correlation between prognostic risk signature and PDCD1. **(F)** Correlation between prognostic risk signature and PDCD1LG2. **(G)** IPS score distribution plot. **(H)** IPS–CTLA4 blocker score distribution plot. **(I)** IPS–PD1/PDL1/PDL2 blocker score distribution plot. **(J)** IPS–CTLA4 and PD1/PDL1/PDL2 blocker score distribution plot.

Given that the information on immunotherapy was not available in the TCGA-LIHC dataset, further analysis was explored for response to immunotherapy. Firstly, the correlation of ICB genes’ (PDCD1, CD274, PDCD1LG2, CTLA-4, HAVCR2, and IDO1) (Kim et al., 2017; Nishino et al., 2017; Zhai et al., 2018) mRNA expression levels to risk score was analyzed (Figure 7A). It was discovered that risk score presented significantly positive correlation with CTLA4 (*r* = 0.15; *p* = 0.0013), HAVCR2 (*r* = 0.19; *p* = 5.2e–05), IDO1 (*r* = 0.093; *p* = 0.05), PDCD1 (*r* = 0.11; *p* = 0.021), and PDCD1LG2 (*r* = 0.12; *p* = 0.0097; Figures 7B–F). To further forecast the immunotherapeutic efficacy of risk score, two subtypes of IPS values (IPS-PD-1/PD-L1/PD-L2 positive and IPS-CTLA-4 positive) were employed as the surrogates of the HCC patients’ responses to immunotherapy. In this predictive model, IPS score, IPS–CTLA4 blocker score, IPS–PD1/PDL1/PDL2 blocker score, and IPS–CTLA4 and PD1/PDL1/PDL2 blocker score were higher in samples with low risk (all *p* < 0.05; Figures 7G–J), suggesting that patients with a low signature score might be suitable for immunotherapy.

### ZC3H13 in Prognostic Prediction, Immune Cell Infiltration, and Immunotherapy

**FIGURE 8:**
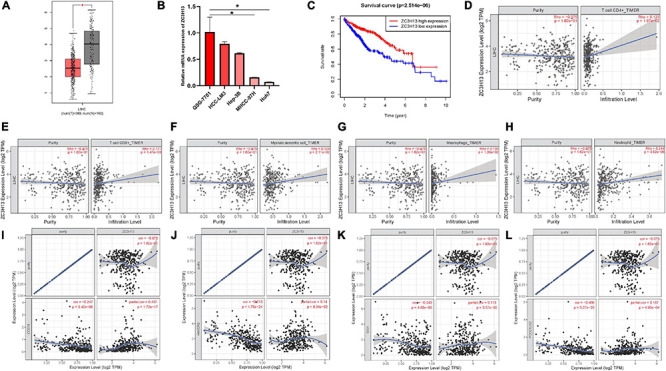
The clinical significance of ZC3H13 in HCC. ZC3H13 are downregulated in HCC samples based on the TCGA dataset **(A)** and cell lines **(B)**, and lower ZC3H13 expression level was significantly correlated with improved prognosis **(C)**. Correlation analysis of prognosis-related genes with infiltrating CD4 + T cells **(D)**, CD8 + T cells **(E)**, dendritic cells **(F)**, macrophages **(G)**, and neutrophils **(H)** using TIMER. The association between the expression levels of ZC3H13 with CD274 **(I)**, HAVCR2 **(J)**, IDO1 **(K)**, and PDCD1LG2 **(L)** using TIMER.

ZC3H13 was the only prognostic m6A regulator with downregulated expression level in tumor samples. Thus, the potential role of ZC3H13 was further explored in subsequent analyses in HCC. The expression levels of ZC3H13 between tumor samples and normal tissues were detected and compared based on TCGA and GTEx data. Compared with tumor samples, the expression level of ZC3H13 was downregulated in adjacent normal tissues (Figure 8A). Taking advantage of qRT-PCR, the expression levels of ZC3H13 were determined in a human hepatic cell line and four distinct HCC cell lines. Consistently, ZC3H13 expression level was lower in tumor cells than in liver cells (Figure 8B). To investigate the prognostic significance of ZC3H13 in HCC, survival curve was analyzed between ZC3H13 high- and low-expressed samples. As a result, the higher expression level of ZC3H13 significantly indicated better prognosis (Figure 8C, *p* = 2.514e–06).

To elucidate the intrinsic relationships between infiltrating immune cells and the expression level of ZC3H13, the correlation of the expression level of ZC3H13 with the immune cell infiltration level was analyzed by using TIMER. Notably, the expression level of ZC3H13 presented significant correlation with CD4 + T cells (*r* = 0.125; *p* = 1.97e–02), CD8 + T cells (*r* = 0.171; *p* = 1.47e–03), myeloid dendritic cells (*r* = 0.124; *p* = 2.11e–02), macrophages (*r* = 0.134; *p* = 1.30e–02), and neutrophils (*r* = 0.244; *p* = 4.62e–06; Figures 8D–H).

Subsequently, the correlation of the expression level of ZC3H13 was analyzed with ICB genes adjusted by tumor purity to reveal the potential roles of ZC3H13 in ICB treatment. The results of TIMER showed that the expression level of ZC3H13 was significantly and positively correlated with CD274 (*r* = 0.437; *p* = 1.73e–17), HAVCR2 (*r* = 0.14; *p* = 9.34e–03), IDO1(*r* = 0.113; *p* = 3.57e–02), and PDCD1LG2 (*r* = 0.187; *p* = 4.90e–04; Figures 8I–L), suggesting the crucial role of ZC3H13 in ICB immunotherapy.

### Role of ZC3H13 in the Context of TIME

**FIGURE 9:**
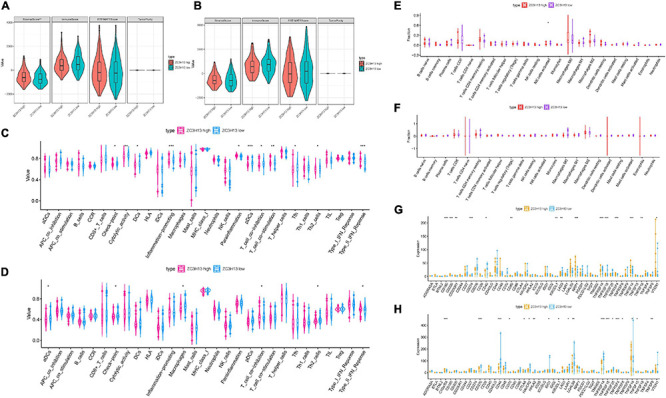
Discrepancy of low and high ZC3H13 expression subgroups in terms of TIME characterization. Comparison of the immune score (ESTIMATE algorithm) between low- and high-ZC3H13 groups in the TCGA cohort **(A)** and ICGC cohort **(B)**. Difference of immune-related signatures between low- and high-ZC3H13 subgroups in the TCGA cohort **(C)** and ICGC cohort **(D)**. Distinction of infiltrating immune cell subpopulations and levels between low- and high-ZC3H13 groups in the TCGA cohort **(E)** and ICGC cohort **(F)**. Comparison of 47 immune checkpoint blockade-related gene expression levels in two ZC3H13 expression subgroups in the TCGA cohort **(G)** and ICGC cohort **(H)**. The asterisks represented the statistical *p* value (*P < 0.05; **P < 0.01; ***P < 0.001).

**FIGURE 10:**
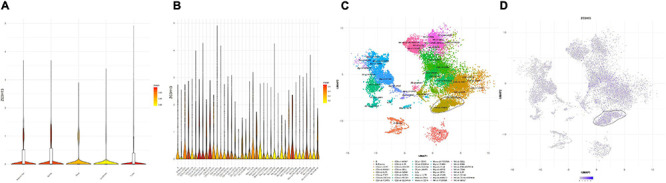
Single-cell RNA sequencing analysis of ZC3H13 abundance in various tissues and immune cell subtypes of HCC patients. **(A)** Analysis of the enrichment of ZC3H13 in tumor, adjacent liver, lymph node, blood, and ascites. **(B)** Analysis of the enrichment of ZC3H13 in immune cell subtypes in tumor tissue. **(C)** UMAP (Uniform Manifold Approximation and Projection) map of immune cells in tumor. **(D)** UMAP map of ZC3H13 expression level in tumors.

To further elucidate the relationship between ZC3H13 and TIME characteristics in HCC, we analyzed the correlation of the ZC3H13 expression value with immune scores and tumor purity (employing the ESTIMATE method), ssGSEA signatures (using GSEABase algorithm), TIC subpopulation and level (*via* CIBERSORT tool), and the expression levels of 47 ICB-associated genes. HCC patients were assigned into low-/high-ZC3H13 subgroups according to the median value of the expression level of ZC3H13. ESTIMATE results suggested that low-ZC3H13 samples obtained significantly lower stromal scores relative to patients in the high-ZC3H13 subgroup in the TCGA cohort but not the ICGC dataset. In terms of immune score, estimate score, and tumor purity, however, there was no remarkable distinction between these two groups (Figures 9A,B). Then, a distinction in immune-associated enrichment was identified between the low- and high-ZC3H13 subgroups. Taken together, the infiltration fraction of Th2 cells, checkpoint, and T-cell co-inhibition significantly increased when risk score declined, whereas IFN-response type II was positively correlated with the expression level of ZC3H13 (Figures 9C,D). The CIBERSORT analysis results of the TCGA cohort showed that the abundance of activated NK cells was significantly higher in patients with low ZC3H13 (Figure 9E). However, there was no remarkable distinction in the ICGC dataset (Figure 9F). Taking advantage of the correlation analysis, we found that three immune check blockade-related genes (i.e., TNFSF14, TNFRSF4, and KIR3DL1) were significantly upregulated, but TNFRSF14 and TNFRSF18 were lower in the high-ZC3H13 group based on two datasets (Figures 9G,H). The results of single-cell sequencing data analysis indicated that ZC3H13 is enriched mostly in tumor samples (Figure 10A). Interestingly, ZC3H13 was predominantly expressed in CXCL13+ CD4+ T cells and FOXP3+ CD4+ T cells (Figures 10B–D). Figures 10C,D show the distribution of ZC3H13 in infiltrating immune cells of TIME. Based on previous findings, CD4-c6-FOXP3 corresponded to regulatory T (Treg) cells (Guo et al., 2018), suggesting that ZC3H13 may serve as an opposing player in HCC progression. Collectively, our findings highlighted that ZC3H13 may play a critical role in TIME context and immunological regulation of HCC.

## Discussion

Hepatocellular carcinoma, one of the most common malignant cancers, is the fourth leading cause of tumor-associated death worldwide. Because of genomic diversity and epigenetic complexity, HCC is characterized by high heterogeneity not only in the clinical but also in the molecular level (Schulze et al., 2016; Cancer Genome Atlas Research Network, 2017; Woo and Kim, 2018). Due to lack of efficient clinical interventions, a sober reality is that the mortality rate of HCC patients is still high (Xu et al., 2021b). Thus, it is urgent to generate powerful tools for prognosis monitoring and clinical outcome prediction, which appears to contribute a novel insight into the decision of clinical management in HCC.

As one of the most abundant forms of endochemical modification in mammals, m6A possesses diverse and crucial biological significance in various pathological processes (He, 2010; Deng et al., 2018; Chen and Wong, 2020; Zhao et al., 2020). Increasing lines of evidence have supported the idea that m6A RNA methylation modulators, which were upregulated or downregulated in numerous categories of malignant tumors, act as promoters or inhibitors of malignancy. Kwok et al. (2017) pointed out that, as a risky predictive prognosis biomolecule of acute myeloid leukemia, the downregulation of the ALKBH5 expression level is significantly correlated with TP53 mutation. METTL3, which is dramatically overexpressed in hepatoblastoma, modulates *b*-catenin to facilitate cancer cell proliferation (Liu et al., 2019). Besides, emerging studies devoted to exploring the key regulatory roles of m6A methylation in TIME focus on elucidating the underlying carcinogenic mechanisms of malignancy. Currently, little is known about the underlying influences of m6A regulators on TIME characterization and immunotherapy in HCC.

In this study, we aimed to elucidate the expression profiling, prognosis predictive performances, and influences on TIME context and ICB therapy of m6A modulators in HCC. We uncovered the differential expression level and correlation of 21 m6A regulators between HCC tissue and normal hepatic specimen based on TCGA-LIHC. The results of the GO analysis showed that overexpressed m6A regulators were mainly enriched in mRNA regulatory procedures, like regulation of mRNA metabolic process and regulation of mRNA stability. Employing consensus clustering, two HCC subtypes were screened based on their m6A RNA modification regulator expression patterns to further reveal their clinical significance and impact on formation of TIME complexity and diversity.

The cluster 1/2 subgroup remarkably affected the OS and distinct clinical parameters of HCC. They presented a significant difference in terms of TIME (i.e., immune score and tumor purity), subpopulation of infiltrating immune cells, and the expression value of ICB-associated genes. Taking advantage of univariate Cox regression followed by LASSO algorithm, a six-gene prognostic risk signature was established, namely, YTHDF1, YTHDF2, IGF2BP3, KIAA1429, METTL3, and ZC3H13. To demonstrate its excellent prognostic performance, the prognostic value was investigated in the TCGA cohort and validated based on the ICGC dataset. We found that risk signature could serve as an independent prognosis predictive indicator through employing both univariable and multivariable COX regression. Besides, a novel nomogram that integrated risk signature and clinicopathological features was generated. GSEA enrichment results indicated the underlying mechanism of risk signature on HCC tumorigenesis and development through mTOR (Mossmann et al., 2018), p53 (Meng et al., 2014; Kong et al., 2017), Wnt (Dai et al., 2019; Hu et al., 2019; Huynh et al., 2019; Li et al., 2019; Tan et al., 2019), and MAPK (Drosten and Barbacid, 2020) signal pathways, among others. Moreover, the as-constructed risk signature was validated to retain a great prognostic value when HCC samples were assigned into subgroups according to clinicopathological variables.

Upon article review, we found that several studies have uncovered the intimate relationship between m6A modification and infiltrating immune cells, which was unable to be clarified by RNA intrinsic metabolic pathways. Dali et al. pointed out that YTHDF1-mediated m6A modification improved TIME CD8 + T cell anticancer efficacy. The inhibition of YTHDF1 enhanced the objective response to ICB (Han et al., 2019). Wang et al. (2019) pointed out that METTL3 bound to the transcripts encoding lysosomal proteases, which enhanced the maturation of dendritic cells (DCs). Therefore, we speculated that the abundance and level of infiltrating immune cells were closely associated with m6A RNA methylation modification. Herein, we validated that clustering results, prognostic risk signature, and the expression level of ZC3H13 were significantly associated with immune infiltration (i.e., dendritic cells). In particular, we observed that the high fraction of activated NK cells suggested better prognosis. Next, we corroborated that clustering results were significantly correlated with proportion of NK cells and m6A-based prognostic signature was significantly associated with NK cell infiltration. Further analysis showed that activated NK cells were negatively associated with ZC3H13 expression, which independently affected OS. These results suggest that m6A regulators might play an undeniable role in the diversity of TIME in HCC mainly through harnessing the activity of NK cells. Furthermore, we discovered that abundance of Tregs was positively correlated with risk score. Consistent results were obtained from single-cell RNA sequencing analysis; ZC3H13 was mainly enriched in Tregs, suggesting that m6A regulators may manipulate the behavior of Tregs to coordinate in the immune network of HCC. Nevertheless, our results are required to be validated in further studies focusing on the underlying mechanism of immunity in HCC development.

With the proposed ICB theory, the administration of immune checkpoint inhibitors has made great breakthroughs in anticancer treatment (Pitt et al., 2016; Llovet et al., 2018; Salik et al., 2020). However, ICB treatment provided few clinical benefits for HCC patients, and less than 33% of patients exhibited objective response to ICB treatment (Liu et al., 2020). Such indicators as tumor mutational burden and expression level of ICB-associated genes were unable to precisely estimate the clinical outcome of immunotherapy. It is therefore of great urgency to recognize predictors for further tailored clinical decision and advance precision treatment (Nishino et al., 2017; Mushtaq et al., 2018; Ng et al., 2020). Numerous studies demonstrated that m6A regulators may play a key role in predicting responsiveness to clinical treatment (Yi et al., 2020; Zhang et al., 2020). Herein, we confirmed that clustering results, m6A-based prognostic signature, and ZC3H13 expression level were significantly correlated with ICB-related genes (i.e., PDCD1). Furthermore, this m6A modulator-based risk signature was positively correlated with ICB-related genes (i.e., CD274), indicating that high-risk samples might be more affected by ICB pathways and present with a better response for immunotherapy. Additionally, increased levels of immunophenoscore, such as IPS–CTLA4 blocker score and IPS–PD1/PDL1/PDL2 blocker score, indirectly suggested the higher tumor immunogenicity for subjects in the low-risk score group. As such, low-risk score patients might be more sensitive to immunotherapy. Notwithstanding, further validation is suggested for these results at larger cohorts and different centers.

Among these m6A regulators, the biological roles of ZC3H13 and KIAA1429 have not been reported in HCC while other m6A regulators (YTHDF1, YTHDF2, IGF2BP3, and METTL3) have been investigated. Besides, the expression level of ZC3H13 but not KIAA1429 could independently affect prognosis. ZC3H13 refers to a CCCH-type zinc finger protein and serves as a vital modulator in the regulation of m6A RNA methylation modification (Wen et al., 2018). Recently, increasing studies have been published focusing on the biological function of ZC3H13 in tumors. For example, a research from Zhu et al. pointed out that ZC3H13 deactivated Ras-ERK to suppress the proliferation and invasion of colorectal cancer (CRC) cells, indicating that ZC3H13 plays an antitumor role in CRC (Zhu et al., 2019). Gewurz et al. (2012) reported that ZC3H13 may act as an oncogene and a key upstream modulator of the NF-kB, which possesses the ability to promote cancer cell invasion and proliferation. Herein, this study was designed to elucidate the prognostic significance and influences on TIME features and ICB treatment of ZC3H13. It was discovered that the expression level of ZC3H13 was significantly downregulated in both cancer tissue and HCC cell lines. Increased level of ZC3H13 expression suggested longer OS time, suggesting that it can serve as a favorable prognosis predictive indicator in HCC. ZC3H13 expression was demonstrated to be positively correlated with the infiltration level of immune cells (i.e., CD8 T cells), which indicated an immune-activated condition, facilitating recognition and elimination of tumor cells and then improving prognosis. The expression level of ZC3H13 significantly and positively correlated with ICB-related genes (i.e., PDCD1LG2), suggesting that samples with high ZC3H13 might be more immunosuppressed by ICB and might obtain benefit from immunotherapeutic treatment. However, the underlying biological function of ZC3H13 in HCC is still unclear, which demands further exploration.

Compared with previous research focusing on the potential role of m6A regulator-mediated methylation in HCC, some superiorities of this work should be noted. Firstly, all HCC samples from the TCGA-LIHC project and the ICGC-LIRI-JP dataset were adopted for comprehensive analysis, and the total specimen size was considerably large. Moreover, the potential players of m6A RNA methylation regulators in the context of TIME (ESTIMATE analysis, ssGSEA algorithm, and CIBERSORT method) and immunotherapeutic prediction (IPS and ICB-related genes) were investigated, which has not been elucidated before this study. In addition, a novel and robust prognostic risk-clinical nomogram plot for clinical practice was developed to predict individual sample OS time quantitatively. Finally, to our knowledge, our study is the first to emphasize on the biological functions of ZC3H13 using comprehensive analysis (prognostic value, immune cell infiltration, and ICB-related key genes) in HCC.

## Conclusion

In summary, the expression pattern, prognostic significance, and impact on TIME context and immunotherapy of m6A RNA methylation regulators were comprehensively analyzed in HCC. The comprehensive analysis of m6A RNA methylation modification could help us understand TIME characterization and facilitate the individualized immunotherapeutic management. However, these findings required validation in further experimental exploration and clinical investigation focusing on the molecular mechanisms of tumor progression and the biological functions of m6A RNA methylation modification in HCC.

## Data Availability Statement

The datasets presented in this study can be found in online repositories. The names of the repository/repositories and accession number(s) can be found in the article/ Supplementary Material.

## Author Contributions

WH designed the overall study and revised the manuscript. QX performed the public data interpretation. HX drafted the manuscript. RD supervised the experiments. NL, RM, and ZQ participated in data collection. YS, ZW, and DW contributed to data analysis. JW and JZ participated in the molecular biology experiments. All the authors read and approved the final manuscript.

## Conflict of Interest

The authors declare that the research was conducted in the absence of any commercial or financial relationships that could be construed as a potential conflict of interest.

## Publisher’s Note

All claims expressed in this article are solely those of the authors and do not necessarily represent those of their affiliated organizations, or those of the publisher, the editors and the reviewers. Any product that may be evaluated in this article, or claim that may be made by its manufacturer, is not guaranteed or endorsed by the publisher.

## Footnotes

1
http://cancergenome.nih.gov

2
https://dcc.icgc.org

3
http://cancergenome.nih.gov/publications/publicationguidelines

4
http://cibersort.stanford.edu/

5
https://cistrome.shinyapps.io/timer/

6
https://tcia.at/home

7
http://cancer-pku.cn:3838/HCC/

## Abbreviations

AFP: alpha-fetoprotein
AUC: area under the curve
CTLA-4: cytotoxic T-lymphocyte antigen 4
CI: confidence interval
CD274: also known as PD-L1
DMEM: Dulbecco’s minimum essential media
FBS: fetal bovine serum
FDR: false discovery rate
FPKM: fragments per kilobase per million
GAPDH: glyceraldehyde-3-phosphate dehydrogenase
GEPIA: gene expression profiling interaction analysis
GO: Gene ontology
GSEA: Gene set enrichment analysis
GTEx: Genotype-Tissue Expression
HCC: hepatocellular carcinoma
HR: hazard ratio
HAVCR2: also known as TIM3
ICB: immune checkpoint blockade
ICGC: International Cancer Genome Consortium
IDO1: indoleamine 2,3-dioxygenase
IPS: immunophenoscore
KEGG: Kyoto Encyclopedia of Genes and Genomes
LASSO: least absolute shrinkage and selection operator
m6A: *N6*-methyladenosine
MAPK: mitogen-activated protein kinase
mTOR: mammalian target of rapamycin
OS: overall survival
PAC: proportion of ambiguous clustering
PD-1: Programmed Cell Death 1
PD-L1: Programmed Cell Death-Ligand 1
PD-L2: Programmed Cell Death-Ligand 2
PDCD1: also known as PD-1
PDCD1LG2: also known as PD-L2
qRT-PCR: quantitative real-time polymerase chain reaction
RNA: ribonucleic acid
ROC: receiver operating characteristic
ssGSEA: single-sample gene set enrichment analysis
TCGA: The Cancer Genome Atlas
TICs: tumor-infiltrating immune cells
TIME: tumor immune microenvironment
TIMER: tumor immune estimation resource
TIM-3: T-cell immunoglobulin domain and mucin domain-containing molecule-3.
