# Supplementary material for: Landscape of Prognostic m6A RNA Methylation Regulators in Hepatocellular Carcinoma to Aid Immunotherapy
Source: Front Cell Dev Biol. 2021 Aug 5;9:669145. doi: 10.3389/fcell.2021.669145 (PMC8375309; doi:10.3389/fcell.2021.669145)
Supplement: Supplementary file 1 [file Data_Sheet_1.DOCX]

**Supplementary Figure Legends:**

**Figure S1:** The boxplot visualizes the abnormally expressed m6A regulators in tumor from ICGC cohort. N represents normal specimen and T represents tumor specimen. Consensus cluster analysis of HCC based on the expression of m6A regulators. (A) The correlation between subgroups when cluster numbers k = 2. (B) Area under cumulative distribution function (CDF) curve when index k ranges from 2 to 10. (C) The relative change in area under the CDF curve for k = 2–9. (D) Heatmap displayed enrichment of 29 immune signatures of these two clusters. Blue represents low expression and red represent high expression. (E) Heatmap of 29 immune signatures and immune scores of these two clusters. Blue represents low expression and red represent high expression.

**Figure S2:** Establishment of the prognostic risk signature. (A) Univariate Cox regression analysis of the m6A regulators. (B-C) The prognostic signature constructed by the minimum criterion of LASSO Cox regression algorithm. (D) Coefficients of six m6A regulators.

**Figure S3:** Confirmation and validation of prognostic risk signature in TCGA(training set) and ICGC (testing set) cohorts. (A-B) Heatmap of the six m6A regulators expression in HCC. The color from red to blue shows a trend from high expression to low expression. (C-D) Distribution of m6A regulators signature risk score. (E-F) The survival status and duration of HCC patients. (G-H) Univariate Cox regression analyses of overall survival. (I-J) Multivariate Cox regression analyses of overall survival.

**Figure S4:** Confirmation of m6A regulators prognostic risk scores in the combined validation group(TCGA and ICGC). (A-D) presents combined cohort findings which are accordant with the previous set results (Figure S3). (E) Univariate Cox proportional hazards analyses of survival in the combined set. (F) Multivariate Cox proportional hazards analyses of survival in the combined set. (G) GSEA delineation of the biological pathways associated with the risk scores of this lncRNA signature utilizing the gene set “c2.cp.kegg.v7.2.symbols”.

**Figure S5:** Correlation of prognostic risk score with immune-related signatures of HCC. Heatmap displayed enrichment of 29 immune signatures of low-/high-risk groups in TCGA cohort(A) and ICGC cohort(B). Blue represents low activity and red represent high activity. Heatmap of 29 immune signatures and immune scores of two different risk score clusters in TCGA cohort(C) and ICGC cohort(D). Blue represents low activity and red represent high activity. (E-F) Comparison of the immune score (ESTIMATE algorithm) between risk score low/high groups in TCGA cohort(E) and ICGC cohort(F).

**Figure S6:** Kaplan–Meier survival analysis for multiple HCC subgroups according to the m6A‐based risk signature stratified by clinical variables. (A-B) Stage. (C-D) T status. (E-F) Gender. (G-H) Tumor grade. (I-J) Age. (K) N status. (L) M status.

Correlation between infiltrating immune cells and m6A-based risk signature.

**Figure S7:** (A)Relationship between this signature and B cells. (B) Relationship between this signature and CD4+T cells. (C) Relationship between this signature and CD8+T cells. (D) Relationship between this signature and Dendritic cells. (E) Relationship between this signature and Macrophages. (F) Relationship between this signature and Neutrophils.
