# Supplementary figures and images for: Landscape of Prognostic m6A RNA Methylation Regulators in Hepatocellular Carcinoma to Aid Immunotherapy
Source: Front Cell Dev Biol. 2021 Aug 5;9:669145. doi: 10.3389/fcell.2021.669145 (PMC8375309; doi:10.3389/fcell.2021.669145)

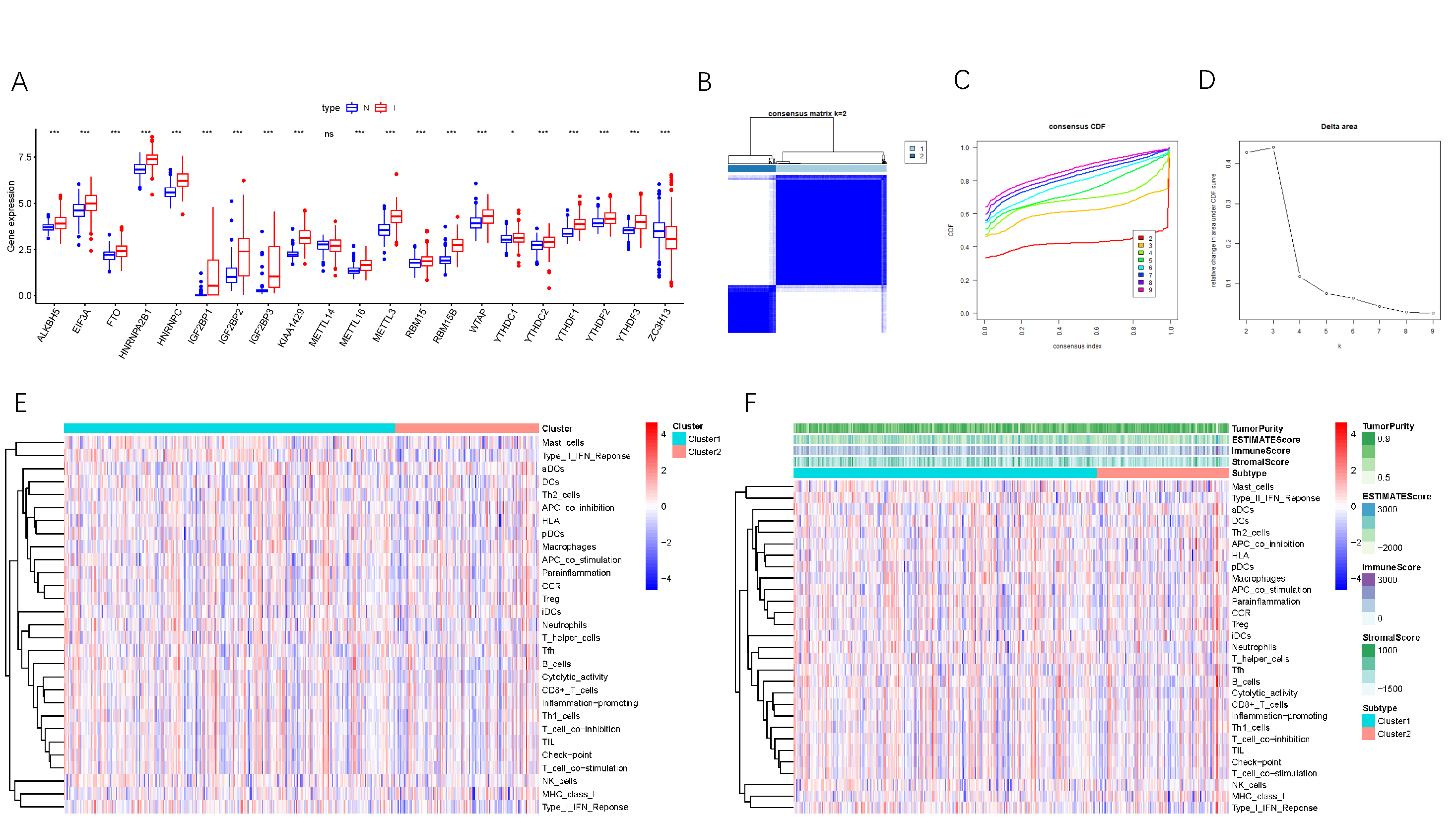

Supplement: Supplementary file 2 [file Image_1.TIFF]

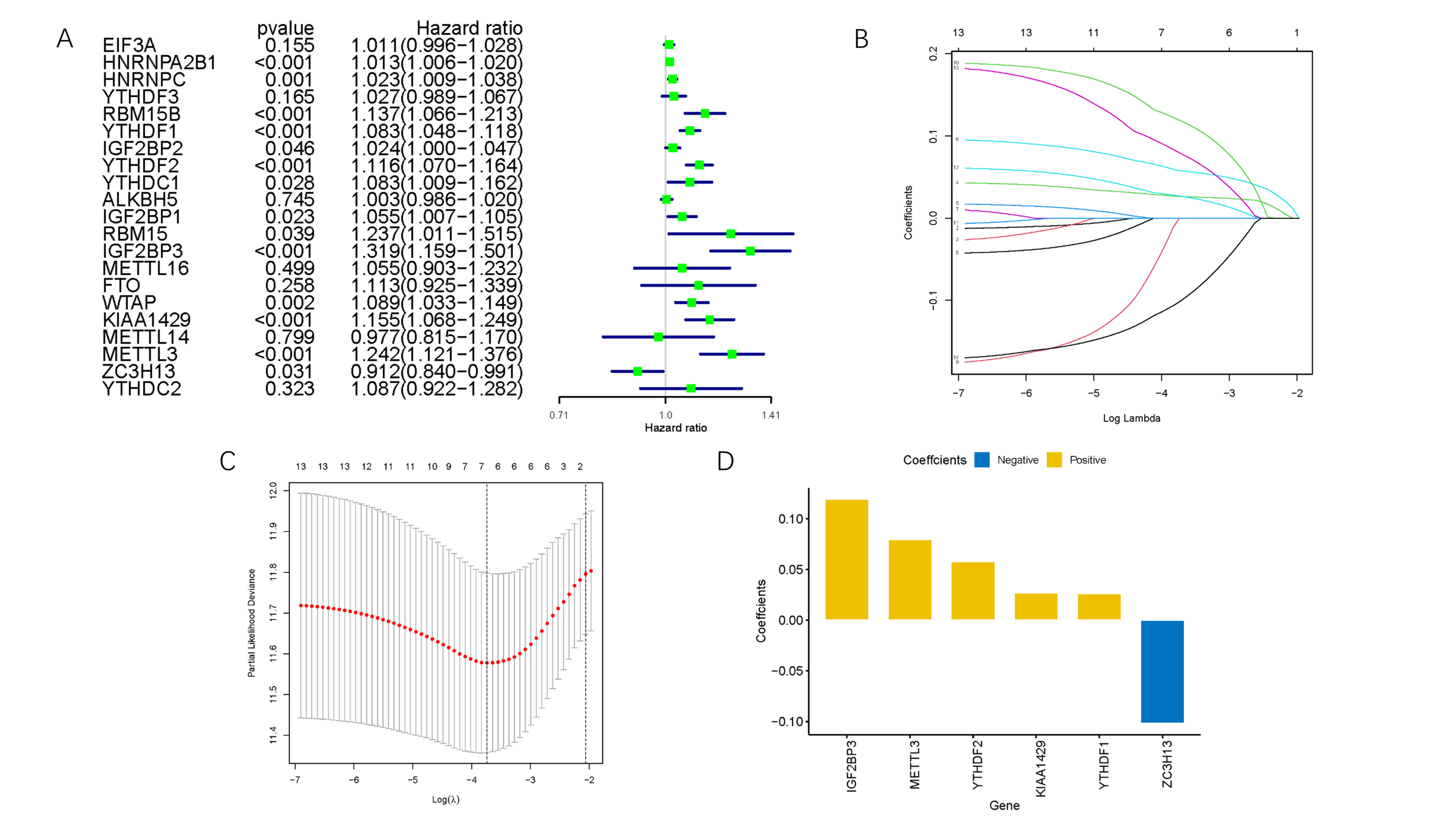

Supplement: Supplementary file 3 [file Image_2.TIF]

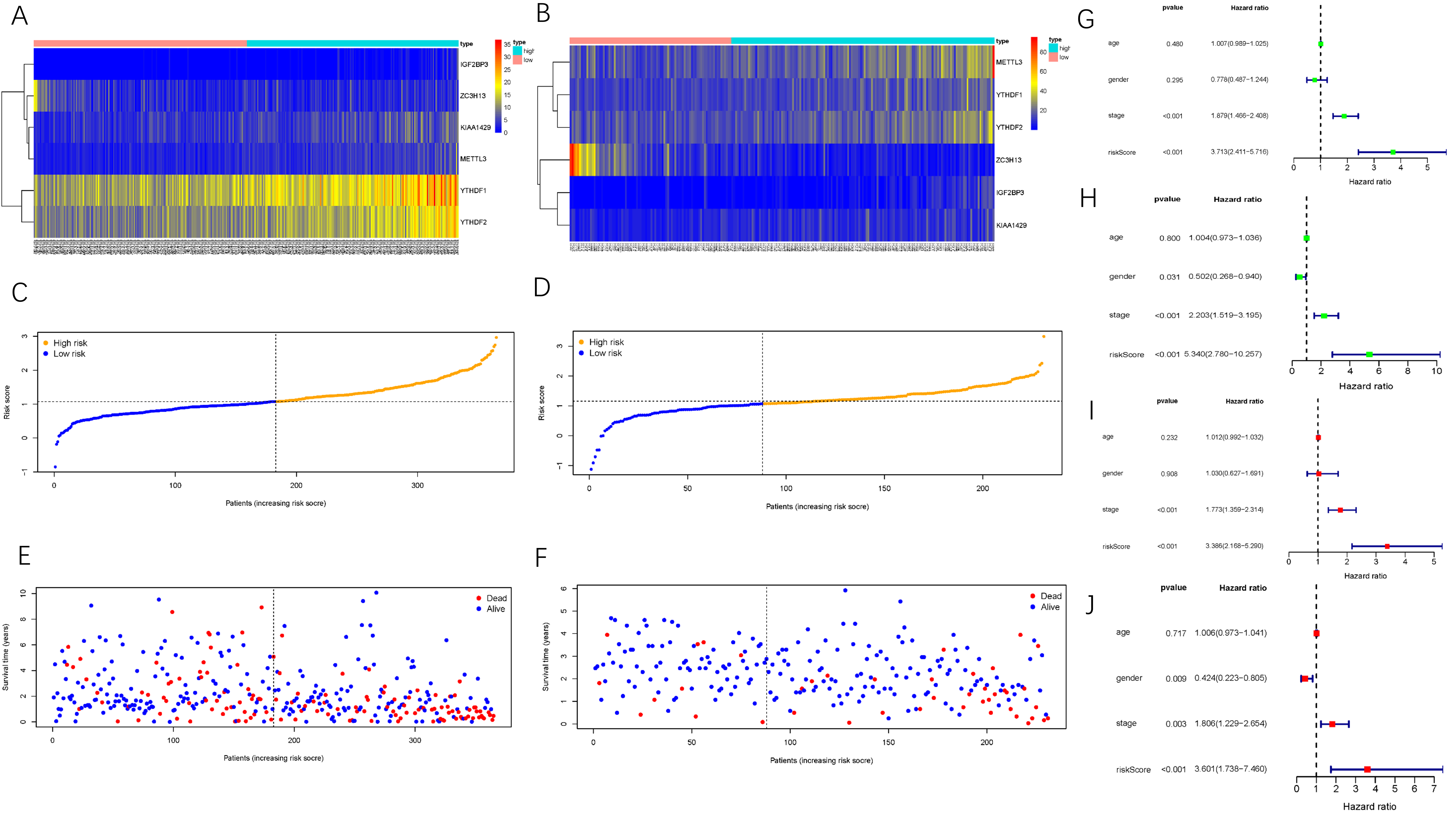

Supplement: Supplementary file 4 [file Image_3.TIF]

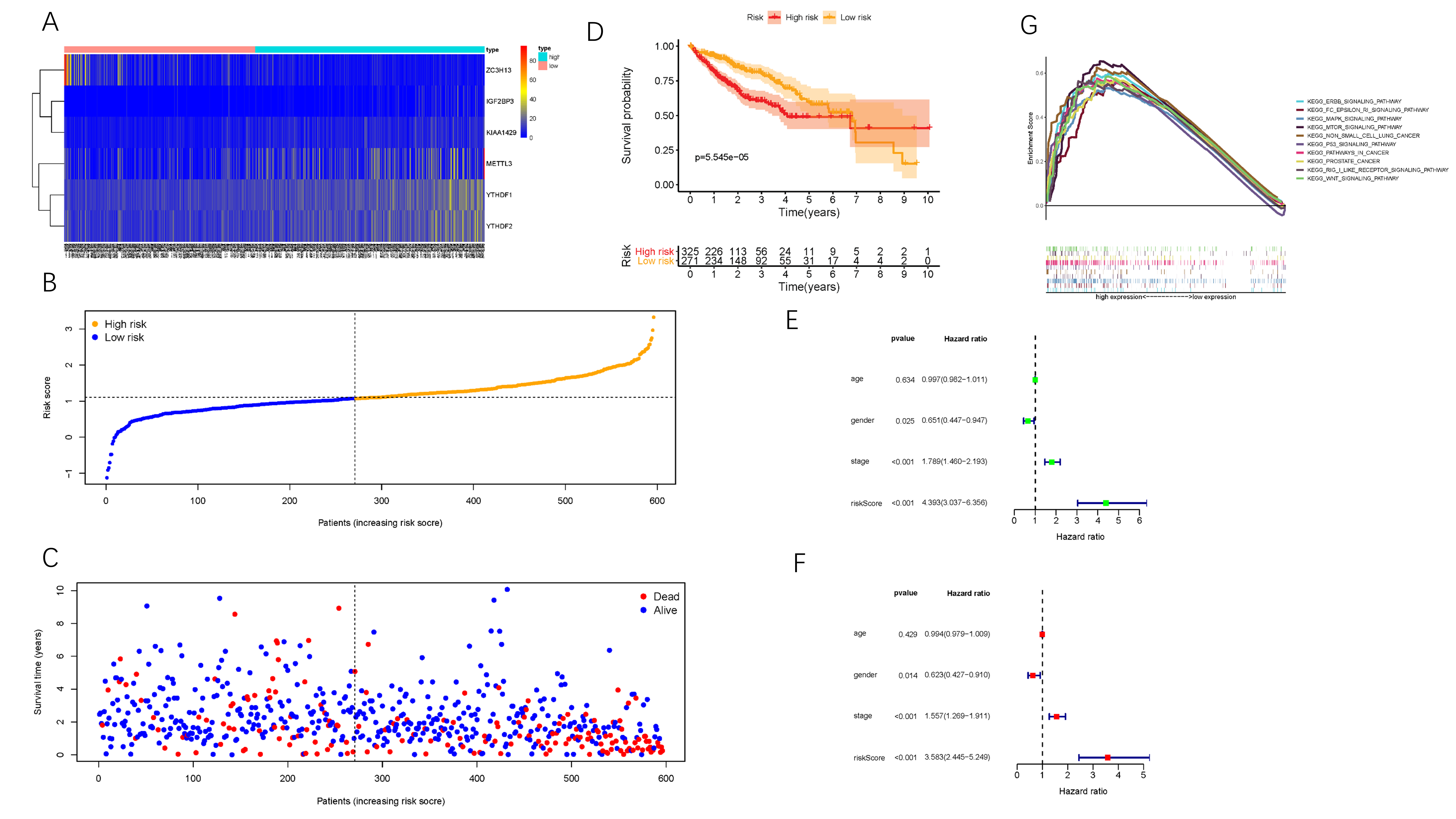

Supplement: Supplementary file 5 [file Image_4.TIF]

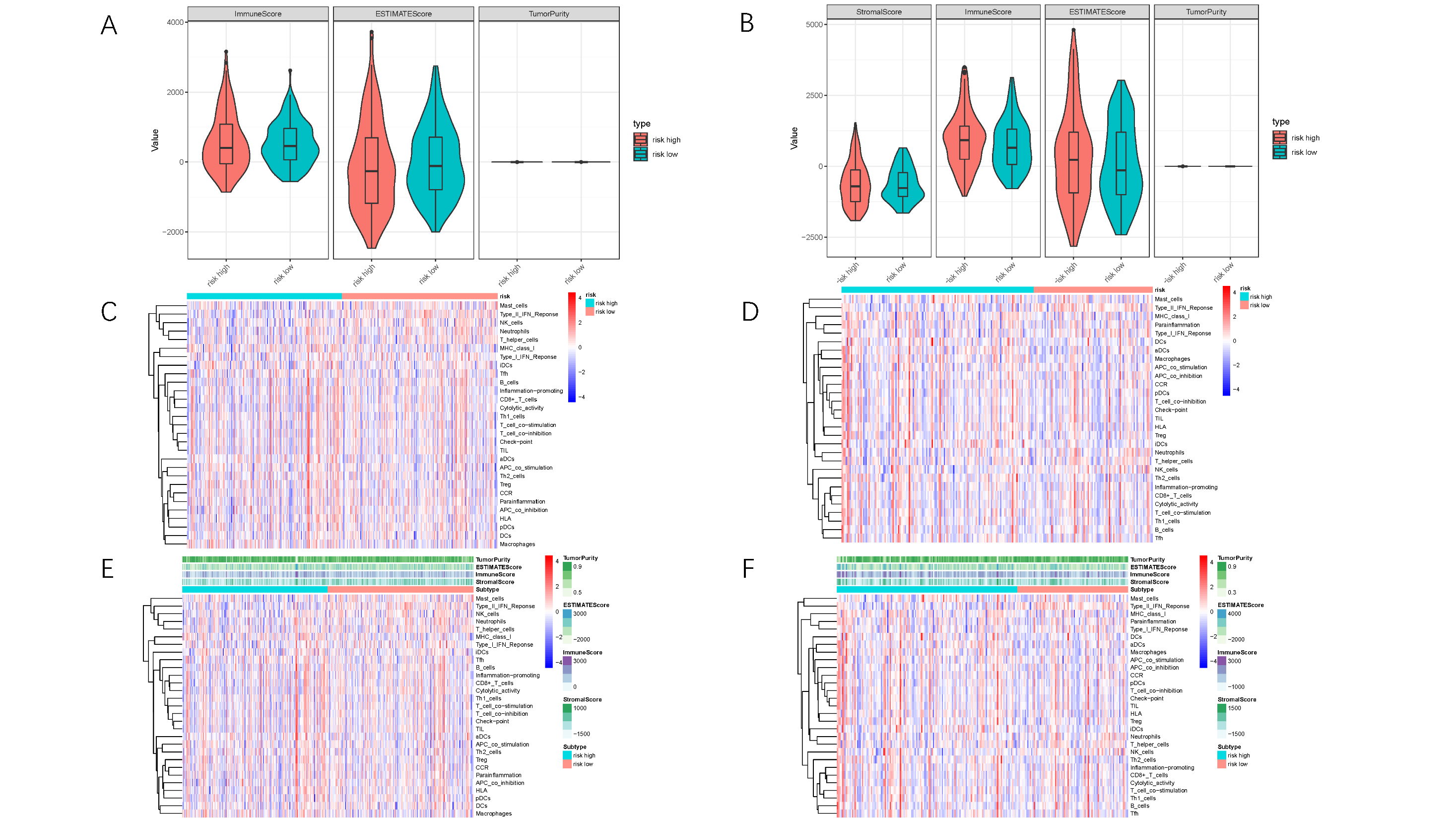

Supplement: Supplementary file 6 [file Image_5.TIFF]

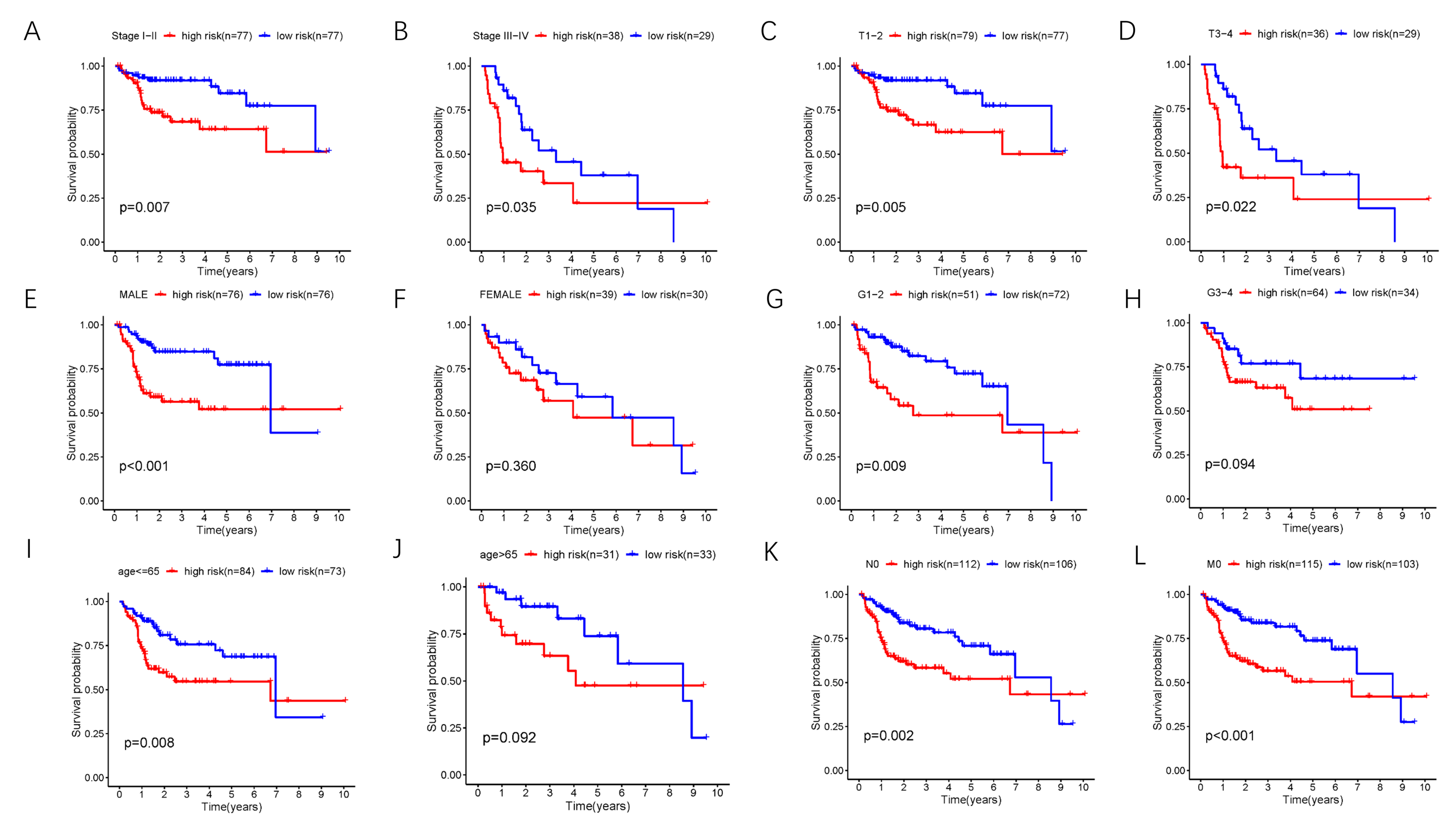

Supplement: Supplementary file 7 [file Image_6.TIF]

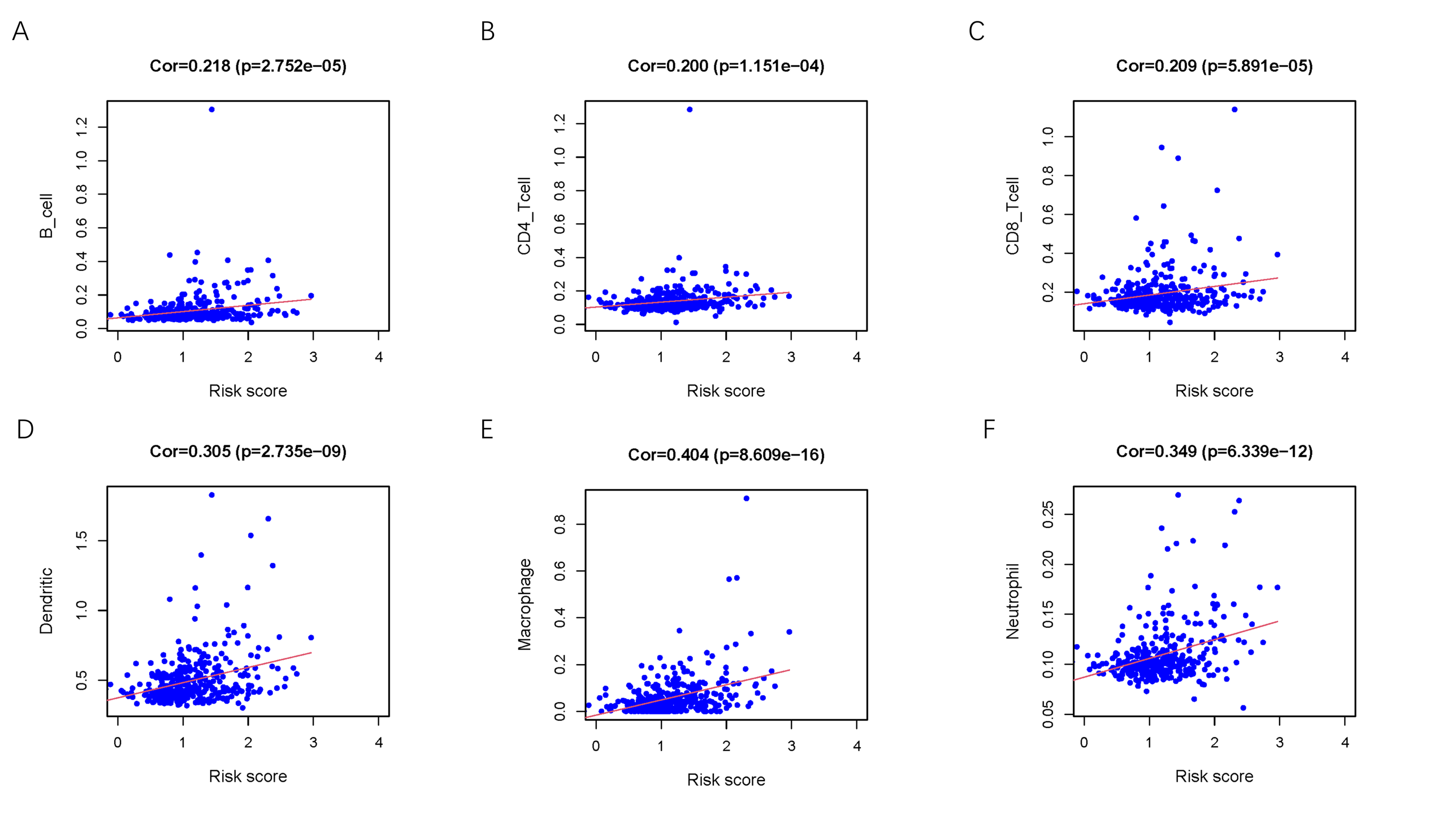

Supplement: Supplementary file 8 [file Image_7.TIF]
